# That's not what you said! Semantic constraints on literal speech

**DOI:** 10.1111/mila.12508

**Published:** 2024-03-15

**Authors:** Sarah A. Fisher

**Affiliations:** ^1^ UCL Department of Political Science and School of Public Policy London UK

**Keywords:** constraint semantics, content semantics, linguistic norms, literal speech, modular semantics, public language, what is said

## Abstract

According to some philosophers, a sentence's semantics can fail to constitute a complete propositional content, imposing mere constraints on such a content. Recently, Daniel Harris has begun developing a formal constraint semantics. He claims that the semantic values of sentences constrain what speakers can literally say with them—and what hearers can know about what was said. However, that claim is undermined by his conception of semantics as the study of a psychological module. I argue instead that semantic constraints should be understood as properties of public languages.

## 1 INTRODUCTION

The question of what semantic properties are—and what the discipline of semantics is the study of—remains an extremely vexed one in contemporary philosophy of language. While it is common to equate a sentence's semantic value with a propositional content, others have argued that sentential semantics merely constrain such contents. Meanwhile, we might conceive of semantics (the discipline) as studying a community's linguistic conventions or individual speakers' psychological processes.

My aim in this article is to argue that constraint semanticists should eschew psychological foundations for their theorising. My strategy will be to critique a proposal put forward by Daniel W. Harris (2022) which seeks to combine both positions. On the one hand, Harris claims that the semantic values of words and sentences constrain what speakers can literally say—and what hearers can know about what was said. On the other hand, he holds that semantic properties are ultimately in the head. As I will show, this is an inherently unstable position. One way out is to adopt a speaker‐relative conception of what is literally said. I will argue, however, that such a conception is of little practical use. A better approach, I suggest, is to understand semantic constraints as properties of public languages, which apply uniformly across linguistic communities and affect what any speaker can literally say with their words.

The structure of the article is as follows. In Section 2 I distinguish constraint semantics from content semantics. In Section 3 I describe Harris's version of constraint semantics. In Section 4 I show that Harris's psychological criterion (which reduces semantic properties to psychological ones) is in tension with his epistemic criterion (according to which hearers can know what speakers can have said, if speaking literally). In Section 5 I argue that it would be a mistake to adopt speaker‐relative semantic constraints on what is literally said (i.e., retaining the psychological criterion and dropping the epistemic one). In Section 6 I argue for semantic constraints that operate independently of individual speakers, being imposed on them by public language conventions (i.e., retaining the epistemic criterion and dropping the psychological one). I conclude in Section 7 that we should understand constraint semantics as the study of a community's linguistic norms, not a speaker's psychological processes.

## 2 CONTENT SEMANTICS AND CONSTRAINT SEMANTICS

The semantic value of a sentence depends in part on the context‐invariant meanings of its lexical constituents (however extensive or emaciated those meanings turn out to be). For example, the semantic value of (1) will depend on the context‐invariant meanings of the expressions, “salmon”, “eat”, and “whitebait”:

(1) Salmon eat whitebait.

It also matters how these lexical constituents are strung together (thus “Salmon eat whitebait” has a different semantic value than “Whitebait eat salmon”, which contains the same elements of surface linguistic form, arranged in a different order). The rules of semantic composition govern how complex sentential values are generated from simple lexical meanings.

Beyond lexical meanings and compositional rules, there is disagreement about which, if any, other ingredients should go into a semantic analysis. Nevertheless, a sentence's semantic value is standardly distinguished from various other things a speaker can pragmatically convey or achieve by using it. Consider, for example, the exchange below:

Irene “I can't understand why there are so many salmon here”.

Rojin “Salmon eat whitebait”.

Rojin's utterance of “Salmon eat whitebait” conveys something like: *It is the abundance of whitebait in the vicinity which explains the presence of so many salmon*. Compare this with Gianni's utterance of the same sentence in the following exchange:

Theo “I'm after some good bait for my fishing trip along the Ayr”.

Gianni “Salmon eat whitebait”.

Here the same sentence is used to convey something quite different, along the lines of: *Whitebait could be suitable for a fishing trip along the Ayr*. Typically, the distinct pieces of information conveyed by Rojin and Gianni are taken to be pragmatic in nature.

How, then should we distinguish semantics from pragmatics? A straightforward way of drawing the boundary would be to identify the semantic value of a sentence with its context‐invariant meaning properties, which remain constant across all uses, while identifying its pragmatic effects with context‐dependent communicative properties.

We might start to doubt this straightforward approach, though, where the context‐invariant meaning of a sentence seems to fall short of a propositional content, as when it contains a context‐sensitive expression.^1^ Consider, for example, the following sentence:

(2) She eats whitebait.

The context‐invariant meaning of the deictic pronoun, “she”, fails to pick out any particular individual independently of its being uttered on a specific occasion. Thus, the context‐invariant meaning of sentence (2) cannot specify *which* individual eats whitebait and, as a result, it is impossible to identify conditions under which the sentence would be true.^2^

At this point, theorists face a choice: Does the semantics of a sentence depend on the extensions of any context‐sensitive expressions it contains? For example, must the semantic value of (2) specify, after all, which individual eats whitebait? Or, alternatively, does it include a mere placeholder for such a referent? This would render its semantics something like “*x* eats whitebait”, where *x* is some individual whose identity must be determined pragmatically in context.

Theorists working in the Kaplanian tradition have tended to take the first route, attributing to sentences *semantic contents*, which depend on the assignment of extensions to context‐sensitive expressions (Kaplan, 1989). An immediate implication of this approach, though, is that semantic analyses will often require some relativisation to context (as when we derive the referent of “she” in specifying the semantic content of (2)). Therefore, the Kaplanian strategy inevitably blurs our proposed boundary between semantics and pragmatics. That boundary can no longer neatly track the distinction between context‐invariant and context‐dependent properties^3^ and must instead be drawn according to a new principle.^4^

The Kaplanian view is countered by those willing to treat semantic representations as intuitively incomplete or underspecified—at least where we are dealing with context‐sensitive elements (Bach, 1987, 2006; Carston, 2002, 2006; Neale, 2004, 2005, 2007a, 2007b; Pietroski, 2006; Schiffer, 2003; Sperber & Wilson, 1995). On this approach, sentences' semantic values act as schematic “blueprints”, which must be filled out in context if we are to arrive at contents equivalent to Kaplan's. Thus, any semantic analysis of sentence (2) would conclude *before* specifying who “she” refers to (although it would require inter alia that the referent be a female individual). These theorists invite us to think of semantic properties as providing mere *constraints* on contents. In this way, it is possible to maintain a straightforward boundary between context‐invariant meaning (semantic) and context‐dependent content (pragmatic).

We might wonder at this point whether the dispute between content semanticists and constraint semanticists is a merely terminological one. Does it really matter whether we assign the label “semantic” to a context‐invariant meaning or a more fully fleshed‐out content? We will see in in what follows how the issue acquires substantive import, depending on which joints of nature we want the semantics‐pragmatics boundary to carve.

## 3 HARRIS'S CONSTRAINT SEMANTICS

Harris (2022) begins to develop a formal constraint semantics. In the next three subsections, I review the features of his view that are most important for my purposes here.

### 3.1 Semantic constraint properties

Harris maintains that the semantic contributions of lexical constituents are their context‐invariant (or “context‐neutral”) meanings, which can be mere constraint properties, rather than full extensions. Consider again the expression “she”. Harris models the constraint property of this expression as requiring the referent to be a female individual,^5^ although *which* female individual remains unspecified.

On Harris's account, the semantics of a sentence is a function of its lexical constituents' context‐invariant meanings, where some of these may be constraint properties. Consider again sentence (2) reproduced below.

(2) She eats whitebait.

Without going into the full technical details of how Harris's compositional rules act on constraint properties, the final semantic analysis of (2) requires that the complete propositional content must intersect the property of eating whitebait with some particular individual who has the property of being female (i.e., the constraint property of “she”). Equivalently, it yields a set of propositions in which different female individuals eat whitebait, generating something like the following semantics for sentence (2):

The property shared by all propositions, *p*, such that, for some female individual, *x*, *p* is the proposition that *x* eats whitebait.^6^

Before proceeding, I note that Harris only presents a formal analysis of overtly context‐sensitive expressions and does not discuss the context‐invariant meanings of open class words like “salmon”, “eat”, and “whitebait”. However, we could assume that their meanings similarly impose constraints on their extensions, filtering out at least some sets of entities as possible referents. For example, the extension of “whitebait” would not include entities that are salmon, trout, or bears (among others). This assumption would capture the idea that an expression's standing meaning constrains its extension, even if it fails to specify a unique extension (in this case, a clearly delineated set of whitebait‐entities). Accordingly, the semantic analysis of a sentence like “Salmon eat whitebait” would require an intersection of the set of entities which is not filtered out by the standing meaning of “whitebait” with the set of entities that salmon eat (a set which might, in turn, remain somewhat underspecified by whatever the semantic constraint properties of “salmon” and “eat” turn out to be).

### 3.2 Modular semantics

Harris's constraint semantics is motivated by his conceptions of semantics and pragmatics as tracking different psychological systems. While pragmatic processes must integrate all sorts of information relevant to what is communicated on a given occasion, Harris takes semantic processing to be modular, in the sense of modularity described by Fodor (1983).^7^ Thus, the semantic module transforms inputs to outputs in a way that is fast and automatic; depends on a limited, proprietary body of information (which Harris calls “semantic competence”); and operates in isolation from the central cognitive processes, which integrate all sorts of other kinds of information required for pragmatic interpretation. In essence, the semantic module is characterised as a discrete psychological system which pairs lexical items with their context‐invariant meanings and composes them in a rule‐governed way, in order to map sentences to semantic representations.

These semantic representations, it is argued, remain incomplete wherever a pragmatic process would be required to fill the gaps—as when sentences include context‐sensitive expressions. Contrasting his view with Kaplanian content semantics, Harris writes:

Content semantics presupposes that all of the information needed to identify the content of a sentence in a context of utterance is available to semantic composition. However, if semantic composition is an informationally encapsulated process, then it does not have access to all of the information that it would need in order to identify lexical expressions' contents. Identifying the contents of semantically underspecified expressions is a process that draws on all manner of central‐cognitive information. If I am right that semantic composition is a modular process and content resolution is a central‐cognitive process, then semantics can deliver only constraints, not contents. (Harris, 2022, p. 313)

A modular account of semantic processing, then, is seen to *entail* a constraint‐based view: If the output of the module is a semantic representation, yet this semantic representation falls short of being a propositional content, then the semantic representation is not a propositional content but (at best) a mere constraint on such a content.

Crucially for Harris, modular semantic processing is taken to be the ultimate target of semantic theorising. In response to the foundational question, “What is the subject matter of compositional semantics?” (Harris, 2022, p. 304) Harris defines it as “the study of a modular component of the mind” (Ibid.). When he claims that “the aim of compositional semantics is to reverse‐engineer language users' semantic competence” (Harris, 2022, p. 309) this is not just because what goes on in the head is a good guide to what external linguistic conventions are. Rather, semantic inquiry is taken to bottom out in specifying those very psychological processes. I will refer to Harris's claim that semantic properties reduce to psychological ones as his *psychological criterion*.

### 3.3 What is literally said

So far, our discussion has skirted around the question of exactly which contents Harris's semantic constraint properties are supposed to constrain. In the event, he claims that they constrain what people can have said, if speaking literally. At the level of lexical constituents, Harris describes the semantic constraint property of a context‐sensitive expression (or “variable”) as “the property that an entity has to have in order for a speaker to use the variable literally to refer to it” (Harris, 2022, p. 321). The semantic value of a sentence, meanwhile, “specifies the range of contents that can be said by using the sentence literally” (Harris, 2022, p. 317).

Harris elaborates on the point by appealing to what competent language‐users can *know* when they encounter bits of language:

Roughly, the semantic value of an expression φ is just what a competent speaker can know about what someone would be saying in uttering φ, assuming they were speaking literally, but without any knowledge about the context or the speaker's intentions. (Harris, 2022, p. 307)

Or again:

If I hear someone utter “it stinks”, and if I assume that they are speaking literally but do not know anything about the context or their referential intentions on this occasion, all that my semantic module tells me about what they have said is that it has this property [possessed by any proposition *p* such that, for some *x*, *p* is the proposition that *x* stinks]. (Harris, 2022, p. 319)

The semantic properties of words and sentences, then, restrict what speakers can use them to literally say; and they entitle other language‐users to know what the speaker can have said, if speaking literally. I will refer to this knowledge‐licensing function of semantic constraints as Harris's *epistemic criterion*.

Unfortunately, Harris does not provide an explicit definition of what it is to speak literally, or to use words or sentences literally. In the discussion that follows, we will gradually home in on exactly what kind of notion he could be appealing to.

## 4 CRITIQUE

I begin by showing how Harris's psychological criterion comes into direct conflict with his epistemic one.

### 4.1 Malapropism

Consider first Harris's discussion of a classic malapropism, in which Yogi Berra utters the sentence “Texas has a lot of electrical votes” (rather than “Texas has a lot of electoral votes”). The effect on the hearer is analysed by Harris as follows:

The hearer's semantic module outputs a representation that gives the hearer evidence that Berra said, of something called “Texas,” that it has a lot of electrical votes. Bringing to bear the resources of central cognition—perhaps unconsciously—the hearer infers that this evidence must not be accurate and, noting the similarity of “electrical” and “electoral,” concludes that Berra meant (and perhaps *said*) [footnote omitted] that Texas has a lot of electoral votes. (Harris, 2022, p. 311)^8^

Harris suggests here that the semantic value of the sentence provides only defeasible evidence of what Berra meant. How can we square this with his claim that semantics constrain what a speaker can literally say?

One way would be to suppose that Berra literally said (but did not mean) that Texas has a lot of *electrical* votes, while meaning (and intending to say) that Texas has a lot of *electoral* votes. Accordingly, a competent hearer would know that, had Berra been speaking literally, he would have said something about *electrical* votes. The hearer concludes, however, that Berra failed to speak literally on this occasion, due to a straightforward performance error.

In practice, I doubt whether Harris would accept this gloss. In footnote 2 of the 2022 paper, as well as in other work, he endorses the view that what speakers say (literally or non‐literally) is ultimately a matter of their intentions. In this respect, he differs from constraint semanticists like Bach, who understand what is said merely as a basis for inferring what the speaker meant (Bach, 1994).

Alternatively, then, Harris might argue that Berra literally said that Texas had a lot of electoral votes, despite having uttered “electrical” rather than “electoral”. But note that, once he allows what was literally said to outstrip semantic constraints in this way, he must drop the claim that semantics constrain what speakers can literally say. The semantic value of a sentence would act only as a clue to what the speaker could have literally said with it.^9^ Opting for this solution would leave semantic representations with very little work to do, and would mark a break with the constraint semantics tradition Harris claimed to be preserving. Therefore, I set this alternative response aside for now.

### 4.2 Stipulation

Harris also imagines a case of stipulation, in which he tells his hearer that he will use the noun “dog” to refer to cats. Reasoning similarly as above, he writes:

[T]he prediction of my view is that when I utter “dog” it will initially seem to my hearer that I am saying something about dogs, since this is what their semantic module indicates, but they may correct for this misleading evidence with a little extra cognitive work. (Harris, 2022, p. 311)

Again, this seems odd: Is the speaker not supposed to be able to know that Harris is saying something about dogs?

In this case too, we could maintain that Harris *literally* said something about dogs, while *non‐literally* saying something about cats. As before, the hearer would know that, had Harris been speaking literally, he would have said something about dogs. In fact, though, Harris is not taken to be speaking literally, having explicitly overruled the dictates of his semantic module.^10^

### 4.3 Divergent semantic processing

In the cases of malapropism and stipulation, we can safely assume that both speaker and hearer process the semantics of “electrical” and “dog” in the same, standard ways (“electrical” means *electrical*, not *electoral*; “dog” means *dog*, not *cat*). They share the relevant semantic competence, which—we are supposing—imposes the same constraints on what each can literally say. Any departures from the ordinary interpretations of the words happen outside the purview of semantics and literal meaning, once other cognitive and motor processes are brought to bear.

But what about situations of genuine divergence in language users' semantic competence? Imagine, for example, that the speaker of (1)—call her Amy—has a semantic module which connects “salmon” with trout and not salmon, while the hearer—call him Bas—has a semantic module which connects “salmon” with salmon and not trout.

(1) Salmon eat whitebait.

According to Harris's psychological criterion, the semantic value of Amy's sentence is whatever representation gets connected to it by her semantic module. Since Amy's semantic module pairs “salmon” with *trout*, that representation will concern trout, not salmon. Therefore, the semantic value of the sentence will constrain her to literally say something about trout not salmon.^11^

Turning now to Harris's epistemic criterion, this states that a competent hearer like Bas can know that Amy would be saying something about salmon in uttering (1), provided she were speaking literally.

Unfortunately, the conjunction of these two verdicts generates a contradiction: If Amy is speaking literally, she is saying something about trout not salmon (according to Harris's psychological criterion); and yet Bas can know she is saying something about salmon not trout (according to Harris's epistemic criterion).

Similar arguments could be run with other sorts of examples—say, where “she” is processed by a speaker's semantic module as constraining the referent to be male rather than female; or where different compositional rules are applied to semantic constraint properties by different speakers. In each case, the psychological and epistemic criteria pull in opposite directions. Thus, Harris's version of constraint semantics is revealed to be inherently unstable.

## 5 SPEAKER‐RELATIVE SEMANTIC CONSTRAINTS

Here is one way out for Harris: Prioritise the psychological criterion and simply accept that semantic constraints are relative to individual speakers.

### 5.1 Relativising what is literally said

On the proposed approach, Amy can use “salmon” to speak literally about trout but not salmon, while Bas can use “salmon” to speak literally about salmon but not trout. (Similarly, the speaker whose semantics for “she” constrain it to refer to male individuals can use that expression to speak literally about male but not female individuals; and vice versa for the speaker whose semantics constrain it to refer to female individuals.)

Note that there is no way to arbitrate the dispute between Amy and Bas. Insofar as semantics is reduced to the study of individual language users' psychological systems, it has nothing to say about whose system should be treated as decisive in a case of conflict (a point we will return to later, in discussing the intuitive normativity of semantic properties). Rather, Amy and Bas are simply operating with different semantic constraints and therefore different possibilities for what each can literally say.

Indeed, the very notion of speaking literally has now collapsed into something like: *Using words and sentences in accordance with the values assigned to them by one's semantic module*. What it means for Amy to be speaking literally, for example, is for her to be using “salmon” to refer to trout, as per the value assigned to that expression by her semantic competence. It then becomes trivial to claim that those semantic values constrain literal speech—by definition, they must do so.

In the meantime, we have ended up dropping or weakening Harris's epistemic criterion (i.e., that competent hearers can know what speakers can have said, if speaking literally). What a language‐user knows on the basis of her semantic interpretation of a sentence is only what they themselves would be constrained to literally say in uttering it. This is not necessarily what another speaker can have said, even if speaking literally. The hearer must therefore assume that (or find out whether) the speaker shares their semantic competence; or otherwise remain agnostic about what the speaker can be literally saying. Thus, while we retain the constraint semanticist's negative claim that semantics can underdetermine what a speaker says, we lose all hope of the speaker's words telling us anything positive about what that content could be.

On the proposed approach, Harris's claim that semantic constraints operate on what speakers can literally say can only be a claim about what a given speaker can literally say, given their particular psychological make‐up, not what *any* speaker can literally say, given the language they are using. Drawing this out explicitly reveals just how weak semantic constraints have become.

It is certainly not obvious from reading Harris (2022) that he intends to make such a weak claim. Nor do I believe this to be the most natural way of understanding his assertion that semantics constrain what speakers can literally say. One contribution of the current discussion, then, is to highlight the implications of taking Harris's psychological criterion seriously. I will now argue that speaker‐relative semantic constraints do little explanatory work and should therefore be eschewed by constraint semanticists.

### 5.2 Explanatory value

We might think that speaker‐relative semantic constraints could help us distinguish between someone's speaking literally and their being ironic, sarcastic, or otherwise figurative. Unlike what is literally said, figurative speech (understood as what is non‐literally said) would remain unconstrained by the speaker's semantic competence. In fact, though, the category of what is non‐literally said would have to include not only figurative speech, but also malapropisms, stipulations, and potentially various other phenomena, where the speaker intends to directly communicate something other than a literal content. The usefulness of the distinction is not entirely clear, then, and it risks collapsing again into just whatever does or does not accord with an individual's semantic competence.^12^

Alternatively, we might think that speaker‐relative constraints have an important role to play in explaining why particular individuals speak and act in the ways they do. For example, if we know that Amy's semantic module links “salmon” with the context‐invariant meaning of trout, her utterance of “Salmon eat whitebait”, in constraining her literal speech to be about trout, may lead us to suppose that she will use whitebait for trout‐fishing (in the absence of evidence that her speech is non‐literal, and depending on various other assumptions we make about her). In contrast, it will not necessarily lead us to suppose that she will use whitebait for salmon‐fishing.

It should be noted, though, that the workings of a speaker's semantic module are *ex hypothesi* inaccessible (not just to other language‐users but also to the speaker's other psychological systems). In practice, then, such applications of the theory are likely to be extremely limited: If we do not know how a speaker's semantic module is configured, we will not know what they can literally say with their words—nor how that could affect their behaviour. Moreover, even where we *can* successfully reverse‐engineer a speaker's semantic processing, it would seem far more parsimonious at that point to explain the relevant aspects of their behaviour by appealing directly to their beliefs (e.g., Amy will use whitebait for trout‐fishing because she believes that “salmon” means *trout*) rather than what they are constrained to literally say (e.g., Amy will use whitebait for trout‐fishing because, in uttering “Salmon eat whitebait” Amy is literally saying something about trout).

In sum, it is unclear what explanatory payoff we get from describing someone's possibilities for literal speech, understood in the speaker‐relative sense, that we do not already get from knowing which meanings they attribute to expressions. Absent other practical applications that have yet to come to light, speaker‐relative semantic constraints on literal speech would seem to have limited value. Moreover, as I argue next, they are incapable of supporting important extant explanatory projects.

### 5.3 The case of content moderation

Why might it be important, in practice, to know what a speaker literally said? Consider a concrete case study of online content moderation.^13^ Social media platforms may intervene on—or “moderate”—a user's speech because it violates the operative terms of service or community standards. This often happens, for example, when a post is deemed to incite violence, perpetrate hate, or spread misinformation. The post might then be prevented from appearing on the platform in the first place, taken down after being published, given a warning label, or algorithmically de‐amplified (meaning that fewer users will see the post in their feed than would otherwise have been the case). Moderation decisions take account of a complex array of factors. One of these is what the speaker can have literally said—in a sense, I suggest, that does not depend on their psychological processing.

Imagine, for example, that someone posts sentence (3) on a platform that prohibits incitements to violence:

(3) Let's meet at parliament at noon tomorrow and attack the prime minister as he leaves.

As a result, the post is removed.

Now suppose that, according to the semantic competence of the author of (3) “attack” means *applaud*; what they intended to propose was applauding, not attacking, the prime minister. Adopting a speaker‐relative conception of semantic constraints on what is literally said, we would then be forced to conclude that the author of (3) literally said something about applauding someone. Yet such a conclusion seems largely redundant in the content moderation case, if not altogether misplaced.

The primary question facing the content moderator is whether and how to intervene on the post.^14^ The user is unlikely to succeed in avoiding moderation of her post by arguing that what she literally said—by her own semantic lights—was perfectly benign. Discovering that the user takes “attack” to mean *applaud* provides no reason for the moderator to reinstate the post. On the contrary, removal is justified by what the words *conventionally* mean. Given what (3) means in English, the user has issued a proposal to attack someone, if speaking literally.^15^ Anything that can be literally said with this sentence would count as inciting violence; and is therefore bound to violate platform rules.^16^

If we hold on to speaker‐relative semantic constraints, we cannot appeal to conventional constraints on what is literally said. There is no sense in which our imagined user issued a proposal to attack someone, even if speaking literally. Thus, a psychologically founded constraint semantics lacks resources for analysing cases of this kind, where content moderation is intuitively justified.^17^

Content moderation depends on human reviewers being able to establish what users might have said, without first having to probe the inner workings of their minds. The case demonstrates the value of Harris's epistemic criterion: It is useful for audiences to know what a sentence can be used to literally say, regardless of the wider context or the speaker's intentions—or, indeed, the inner workings of their linguistic processing systems—in moderating rule‐violating speech. As we have seen, speaker‐relative semantic constraints are inadequate to that task.

The general lesson is that we get real explanatory pay‐off from conceiving of such constraints as conventions that operate—from the outside, as it were—on what any member of a linguistic community can literally say with their words. This is why I believe constraint semanticists should jettison Harris's psychological criterion and uphold the epistemic one. Before concluding, I will briefly sketch the contours of the resulting view I have in mind.

## 6 CONVENTIONAL SEMANTIC CONSTRAINTS

The position I want to defend is that semantic constraints should be understood as linguistic conventions or norms, which affect what any speaker can literally say with their words. In doing so, I follow in the footsteps of many philosophers who have resisted attempts to reduce meaning or saying to individual speakers' psychologies or behaviours—see, for example, rebuttals of Davidson's (1986) attempt to divorce the literal from the conventional, including those made by Dummett (1986), Green (2001), Reimer (2004), and Stainton (2016). While I lack the space here to engage closely with each of their views, many of the general arguments they put forward could be carried across to provide further support for my more specific claim—namely that, in answer to Harris's foundational question, compositional semantics should be understood as the study of a set of external rules (not a modular component of the mind); and its ultimate aim is to reverse‐engineer permissible operations in a public language (not a language user's semantic competence).

### 6.1 Divergent semantic processing (revisited)

Consider again our imagined speaker, Amy, who, believing that “salmon” means *trout*, utters the sentence “Salmon eat whitebait” intending to communicate that trout eat whitebait. I believe a natural gloss of the situation is as follows: What Amy intends to convey to Bas is that *trout* eat whitebait but what she literally says is that *salmon* eat whitebait.^18^ I suggest that there is a very straightforward reason for this—namely that, in English, “salmon” refers to salmon, not trout.

One might object along the following lines. When Amy is described as having literally said that salmon eat whitebait, this only sounds natural because “said” is being used in the sense of “uttered”; we are simply quoting the words she used, rather than giving an interpretation of them. So, the case should really be described thus: Amy intended to communicate that trout eat whitebait but the sentence she uttered was “Salmon eat whitebait”. And, while this is clearly right, Harris is interested in a sense of (literally) saying that does not merely involve quoting a speaker's words but giving them a (literal) interpretation.

In response to this objection, I too am interested in a substantive sense of literally saying. My claim is just that the literal interpretation we give should be in line with the words' conventional meanings.

### 6.2 Linguistic norms

I am proposing a conception of compositional semantics as specifying conventional logical operations performed on lexical items' conventional context‐invariant meanings. In principle, such a study can proceed independently of specifying psychological operations; it investigates public languages, which are the upshots of complex social interactions between individuals and across historical time.^19^

One question immediately raised by the proposed approach is *which* linguistic conventions or norms are relevant to the determination of semantic constraints? While I cannot hope to provide a comprehensive answer here, I note the importance of distinguishing semantic norms from other kinds of (linguistic and non‐linguistic) norms. For example, the fact that the expression “she” can be used to refer to a female individual is a semantic norm, while the fact that it is pronounced /ʃi:/ is not. Nor is the emerging convention to use “she” only once the individual being referred to has declared her preferred pronouns. As a rough first pass at delineating those norms which are semantic, I suggest that they would prominently include dictionary definitions of words, codified compositional rules, and practices of deference to experts (for example, in relation to technical terms). However, semantic norms would not concern matters of articulation, syntactic well‐formedness, or (non‐meaning‐constituting) rules governing when it is socially appropriate to use one word rather than another.

The individuation of languages and linguistic communities will also require a degree of care and sophistication. For example, consider again Harris's case of stipulation, in which he tells his hearer that he will use the noun “dog” to refer to *cats*. Suppose the hearer agrees to follow his usage. Arguably, this introduces a new convention for the sub‐community constituted by Harris and the hearer. Within that sub‐community, it might be possible to literally say things about *cats* by using the word “dog”. More generally, speakers of a language might adopt somewhat different conventions among different groups of interlocutors. Likewise, a single public language may have several different dialects, requiring some semantic norms to be specified relative to sub‐communities of speakers. These considerations suggest a nuanced picture, in which individual speakers can be part of multiple nested and overlapping linguistic communities.

Finally, more will ultimately need to be said about what makes a language‐user count as competent, insofar as such a person is capable of reliably tracking semantic norms. What I have in mind by appealing to competence here is that only those who are sufficiently familiar with a language's conventions are likely to be good judges of the operative semantic norms.

The key feature of the view just sketched is that it makes room for semantic meaning to be determined communally rather than individually. It locates the possibilities for literal speech at the level of public language, rather than individual speakers. As such, it enables what is literally said to outstrip what accords with an individual speaker's semantic competence. In this way, semantic constraints get their bite back: They impose themselves on language‐users from the outside, rather than being a matter of mere internal coherence.

### 6.3 Normativity

While semanticists are often engaged in providing a *descriptive* account of the constraints imposed on literal meaning, these constraints are also plausibly the sources of many everyday evaluative judgements (as should be expected when we are dealing with a species of social norms). For instance, if we find out that a speaker thinks “salmon” refers to *trout*, we commonly judge her to be wrong about this.^20^ The sense in which they are wrong is not that their expressed belief about the meaning of the word contradicts their actual modular semantic competence (which would be the only ground for normativity in a speaker‐relative account). Rather, the problem is that the speaker's semantic competence is out of step with what the word is used to mean in the wider linguistic community.

What this shows is that semantics imposes important normative constraints on speakers (as has been argued by Dummett (1986) and Stainton (2016) among others). Not only does a speaker, as a matter of fact, literally say something about salmon when she uses the word “salmon” but there is also a sense in which she *should* use “salmon” to mean *salmon*, as a participant in the community of linguistic practice.^21^ Thus, at least on many occasions when we talk about what someone literally said, we are not merely stating a fact about the status of their action but making a *normative* appeal to how words ought to be used, according to linguistic standards. This normative function of semantic constraints is one which Harris's psychological account is unable to accommodate. The alternative approach I have outlined, however, is well‐placed to do so.

The point about normativity connects importantly to the motivation for distinguishing between context‐invariant semantic properties and context‐dependent pragmatic properties (beyond mere tidy philosophical categorisation). Harris argued for this division on the basis that it tracked the psychological systems underlying each category. One might worry then, that jettisoning an essentially psychological definition of semantics risks throwing out our reason for drawing the semantics‐pragmatics boundary in the proposed way. However, this is not the case. Relocating semantics from the heads of speakers to the language of the community suggests a different, more socially‐oriented argument for separating context‐invariant and context‐dependent properties of speech. Specifically, the way societies regulate speech and discourse often requires appeal to semantically constrained literal meaning, understood speaker‐independently.

We have seen an example of this in the case of online content moderation. Deciding when and how to intervene on user speech depends, at least in part, on what a speaker can have been saying, if speaking literally. And that does not plausibly depend on finding out how a speaker's semantic processes are configured. Rather, it depends on what words and sentences conventionally mean in the language used.

The more general lesson is that, given the enormously influential role of speech and testimony in human society—leading people to believe, decide, and act in the ways they do—we frequently need to invoke a shared notion of what was literally said in deciding how to assess and react to people's public utterances. This is true regardless of whether the speech occurs online or offline, in formal institutional settings or in day‐to‐day social interactions.

Of course, there remains a role in our theorising for analysing individual‐level linguistic processing (and, for all I say here, the processing of semantic phenomena might still turn out to be modular in structure). However, the psychological phenomena involved would now be understood as attempts to latch onto external linguistic conventions, not the primary, foundational, object of semantic study.^22^ In slogan form, semantics is not semantic processing. The purpose of this section has been to point to an alternative, which locates semantics in the conventions of the linguistic community.

## 7 CONCLUSION

I have argued in this article that Harris's constraint semantics, purporting as it does to model the workings of a psychological module, cannot do justice to the substantive sense in which semantic constraints are supposed to affect what any speaker of a language can literally say with their words. Instead, constraint semanticists ought to adopt a view of semantics as, foundationally, the study of norms which exist at the level of the linguistic community. Such an approach is needed if we are to give plausible analyses of real‐world judgements about the meanings of words and sentences.

## Data Availability

There is no data available.

## Footnotes

^1^
Arguably, the problem does not only arise for overtly context‐sensitive expressions like indexicals and demonstratives, but also for quantifiers (Neale, 2007b; Stanley & Szabo, 2000), gradable adjectives (Kennedy & McNally, 2005; Stanley, 2002), and perhaps even all open class words (Carston, 2013; Travis, 2008).

^2^
Although we might be able to identify some conditions under which it would be false, and this might be sufficient for (2) to count as truth‐evaluable in a broad sense. For further discussion, see Fisher (2021).

^3^
Although see Borg (2004, 2012) for an attempt to square the circle.

^4^
Thus, some have attempted to link semantics to intuitive truth‐conditions—see, for example, Stanley and Szabo (2000).

^5^
Formally, *λx*_e_. *x* is female.

^6^
One could imagine a different version of constraint semantics, according to which the meanings of lexical constituents get pragmatically enriched *prior to* composition (as proposed by Recanati, 2004, 2010). However, there is no reason to suppose that semantic composition prior to pragmatic enrichment is *impossible*—indeed, Harris's formal theory refutes this. And, insofar as one thinks that composition prior to pragmatic enrichment merely produces the *wrong* content—that is, one which was not intended or communicated—this is largely orthogonal to Harris's claim that a sentence's semantic value constrains what a speaker would have been saying, if speaking *literally*.

^7^
But note that Fodor did not himself commit to *semantic* processing being modular.

^8^
In the footnote, Harris cites Unnsteinsson's (2017) argument that what a speaker says with a malapropism may be their intended meaning. According to Unnsteinsson: When speakers perform a speech error and produce a syntactically well‐formed sentence different from the one they intended, they don't thereby *say* what that sentence is normally taken to say. They simply produce a sentence that could have been uttered—if they had had the requisite communicative intentions—to say what it is normally taken to say. (Unnsteinsson, 2017, pp. 459–460) I discuss below the implications of adopting this kind of intentionalist stance towards what is (literally) said.

^9^
In personal communication, Harris indicates that something like “clue semantics” could indeed be a more accurate label for his view.

^10^
Again, I suspect that Harris would ultimately reject this gloss, for the same reasons as discussed in the previous subsection—and with the same effect of undermining his claim that semantics constrain what speakers can literally say.

^11^
Could Harris get around this conclusion by appeal to externalism about mental content? Perhaps. Content externalism would guarantee that every speaker's semantic module paired “salmon” with a context‐invariant meaning that picked out the relevant natural kind—even if a speaker like Amy were to use the word in a different way. The kind of divergence envisaged above would then be ruled out of court. I doubt whether Harris would wish to be committed to that position (not least because it would drive a wedge between semantic competence and linguistic behaviour, making it impossible to explain the latter in terms of the former). However, it would be interesting if content externalism turned out to be an implication of his account.

^12^
A reviewer for this journal suggests that speaker‐relative semantic constraints could help us distinguish between what a speaker communicates directly and what they communicate indirectly. I do not think this can be right. Indeed, figurative speech is a counterexample, being non‐literal but direct (since the speaker directly communicates a non‐literal content instead of the literal one) in contrast with conversational implicatures, which are non‐literal and indirect (being conveyed in addition to, not instead of, a directly communicated literal content). Therefore, the literal/non‐literal distinction cuts across the direct/indirect one.

^13^
For related discussion of strict linguistic liability in legal and contractual contexts, see Stainton (2016, p. 25) and Borg (2019, section 4).

^14^
Albeit a secondary question might concern whether to apply sanctions to the user, like restricting their access to a platform. Insofar as the speaker had no ill intent, we might not wish them to undergo such sanctions. Still, as per the previous subsection, we can easily accommodate this point by acknowledging the speaker's beliefs about the meaning of “attack”. There is nothing to be gained by appealing additionally to what was literally said.

^15^
Of course, without wider contextual information, a moderator would not know which prime minister the user is referring to, nor which day, which parliament, and so forth. These are the kinds of considerations that have led constraint semanticists to argue that sentential semantics fall short of constituting complete contents. However, I take it that they would deny that “attack” could mean *applaud*.

^16^
To be sure, semantic facts will generally not be *sufficient* to guide moderation decisions; moderators will need to consider various aspects of the wider context in assessing which speech acts were performed. Semantic facts will often, however, be *necessary* for that process.

^17^
Could it be argued that, although the user literally proposed to *applaud* the prime minister, content moderation is justified by the fact that most of the audience would understand the post as a proposal to *attack*? I think this is nearly right. The pertinent question, though, is *why* most speakers would adopt that interpretation; the answer, I suggest, is that it is the one secured by English language conventions, which are the source of semantic constraints.

^18^
Devitt (2021, Chap. 5) reports the opposite intuition in such a case. He argues that the speaker in question does not count as participating in the relevant linguistic conventions. However, insofar as Amy is attempting to speak the language of the community, and would be disposed to adjust her usage upon discovering the conventional meaning of a word, it seems to me that she *is* participating.

^19^
This is not to say that there could be semantic phenomena without the existence of any bearers of psychological states and processes. Dummett makes a similar point: “Of course, [words] do not have [meanings] intrinsically, and hence independently of anything human beings do. They have them in virtue of belonging to the language, and hence in virtue of the existence of a social practice. But they have them independently of any particular speakers” (Dummett, 1986, p. 473). My point, then, is that semantics cannot be reduced to an *individual's* psychology.

^20^
See Reiland (2023) for further discussion of linguistic mistakes. The broad thrust of the argument presented there is complementary to the one I put forward here.

^21^
Of course, this normative injunction may be overridden by other considerations. For example, if the speaker has blamelessly received false testimony about salmon, then perhaps it is false to say that she should use the term correctly, all things considered.

^22^
Dummett makes a similar point when he notes that “a common language is related to an idiolect essentially as the rules of a game are related to a player's beliefs about what they are” (1986, p. 469).

## References

[mila12508-bib-0001] Bach, K. (1987). Thought and reference. Clarendon Press.

[mila12508-bib-0002] Bach, K. (1994). Semantic slack: What is said and more. In S. L. Tsohatzidis (Ed.), Foundations of speech act theory: Philosophical and linguistic perspectives (pp. 267–291). Routledge.

[mila12508-bib-0003] Bach, K. (2006). The excluded middle: Semantic minimalism without minimal propositions. Philosophy and Phenomenological Research, 73(2), 435–442.

[mila12508-bib-0004] Borg, E. (2004). Minimal semantics. Clarendon Press.

[mila12508-bib-0005] Borg, E. (2012). Semantics without pragmatics. In K. Allan & K. Jaszczolt (Eds.), Cambridge handbook of pragmatics (pp. 513–528). Cambridge University Press.

[mila12508-bib-0006] Borg, E. (2019). Explanatory roles for minimal content. Noûs, 53, 513–539.

[mila12508-bib-0007] Carston, R. (2002). Thoughts and utterances: The pragmatics of explicit communication. Blackwell.

[mila12508-bib-0008] Carston, R. (2006). Relevance theory and the saying/implying distinction. In L. R. Horn & G. Ward (Eds.), The handbook of pragmatics (pp. 633–656). Blackwell.

[mila12508-bib-0009] Carston, R. (2013). Word meaning, what is said, and explicature. In C. Penco & F. Domaneschi (Eds.), What is said and what is not (pp. 175–204). CSLI Publications.

[mila12508-bib-0010] Davidson, D. (1986). A nice derangement of epitaphs. In E. Lepore (Ed.), Truth and interpretation: Perspectives on the philosophy of Donald Davidson (pp. 433–446). Blackwell.

[mila12508-bib-0011] Devitt, M. (2021). Overlooking conventions: The trouble with linguistic pragmatism. Springer International Publishing.

[mila12508-bib-0012] Dummett, M. (1986). ‘A nice derangement of epitaphs’: Some comments on Davidson and Hacking. In E. Lepore (Ed.), Truth and interpretation: Perspectives on the philosophy of Donald Davidson (pp. 459–476). Blackwell.

[mila12508-bib-0013] Fisher, S. A. (2021). Reassessing truth‐evaluability in the minimalism‐contextualism debate. Synthese, 198, 2765–2782.

[mila12508-bib-0014] Fodor, J. A. (1983). The modularity of mind: An essay on faculty psychology. MIT Press.

[mila12508-bib-0015] Green, K. (2001). Davidson's derangement: Of the conceptual priority of language. Dialectica, 55(3), 239–258.

[mila12508-bib-0016] Harris, D. W. (2022). Semantics without semantic content. Mind & Language, 37, 304–328.

[mila12508-bib-0017] Kaplan, D. (1989). Demonstratives: An essay on the semantics, logic, metaphysics and epistemology of demonstratives and other indexicals. In J. Almog , J. Perry , & H. Wettstein (Eds.), Themes from Kaplan (pp. 481–563). Oxford University Press.

[mila12508-bib-0018] Kennedy, C. , & McNally, L. (2005). Scale structure, degree modification, and the semantics of gradable predicates. Language, 81(2), 345–381.

[mila12508-bib-0019] Neale, S. (2004). This, that, and the other. In A. Bezuidenhout & M. Reimer (Eds.), Descriptions and beyond (pp. 68–182). Oxford University Press.

[mila12508-bib-0020] Neale, S. (2005). Pragmatism and binding. In Z. G. Szabo (Ed.), Semantics versus pragmatics (pp. 165–285). Oxford University Press.

[mila12508-bib-0021] Neale, S. (2007a). Heavy hands, magic, and scene‐reading traps. European Journal of Analytic Philosophy, 3(2), 77–132.

[mila12508-bib-0022] Neale, S. (2007b). On location. In M. O'Rourke & C. Washington (Eds.), Essays on the philosophy of John Perry (pp. 251–393). MIT Press.

[mila12508-bib-0023] Pietroski, P. M. (2006). Character before content. In J. J. Thomson & A. Byrne (Eds.), Content and modality: Themes from the philosophy of Robert Stalnaker (pp. 34–60). Oxford University Press.

[mila12508-bib-0024] Recanati, F. (2004). Literal meaning. Cambridge University Press.

[mila12508-bib-0025] Recanati, F. (2010). Truth‐conditional pragmatics. Oxford University Press.

[mila12508-bib-0026] Reiland, I. (2023). Linguistic mistakes. Erkenntnis, 88, 2191–2206.

[mila12508-bib-0027] Reimer, M. (2004). What malapropisms mean: A reply to Donald Davidson. Erkenntnis, 60, 317–334.

[mila12508-bib-0028] Schiffer, S. (2003). The things we mean. Clarendon Press.

[mila12508-bib-0029] Sperber, D. , & Wilson, D. (1995). Relevance: Communication and cognition (2nd ed.). Blackwell.

[mila12508-bib-0030] Stainton, R. (2016). A deranged argument against public languages. Inquiry, 59(1), 6–32.

[mila12508-bib-0031] Stanley, J. (2002). Nominal restriction. In G. Preyer & G. Peter (Eds.), Logical form and language (pp. 365–388). Clarendon Press.

[mila12508-bib-0032] Stanley, J. , & Szabo, Z. G. (2000). On quantifier domain restriction. Mind & Language, 15(2), 219–261.

[mila12508-bib-0033] Travis, C. (2008). Occasion‐sensitivity: Selected essays. Oxford University Press.

[mila12508-bib-0034] Unnsteinsson, E. (2017). A Gricean theory of malaprops. Mind & Language, 32(4), 446–462.

